# Targeted Assembly of Short Sequence Reads

**DOI:** 10.1371/journal.pone.0019816

**Published:** 2011-05-11

**Authors:** René L. Warren, Robert A. Holt

**Affiliations:** 1 Genome Sciences Centre, British Columbia Cancer Agency, Vancouver, British Columbia, Canada; 2 Department of Molecular Biology and Biochemistry, Simon Fraser University, Burnaby, British Columbia, Canada; Université Paris-Sud, France

## Abstract

As next-generation sequence (NGS) production continues to increase, analysis is becoming a significant bottleneck. However, in situations where information is required only for specific sequence variants, it is not necessary to assemble or align whole genome data sets in their entirety. Rather, NGS data sets can be mined for the presence of sequence variants of interest by localized assembly, which is a faster, easier, and more accurate approach. We present TASR, a streamlined assembler that interrogates very large NGS data sets for the presence of specific variants by only considering reads within the sequence space of input target sequences provided by the user. The NGS data set is searched for reads with an exact match to all possible short words within the target sequence, and these reads are then assembled stringently to generate a consensus of the target and flanking sequence. Typically, variants of a particular locus are provided as different target sequences, and the presence of the variant in the data set being interrogated is revealed by a successful assembly outcome. However, TASR can also be used to find unknown sequences that flank a given target. We demonstrate that TASR has utility in finding or confirming genomic mutations, polymorphisms, fusions and integration events. Targeted assembly is a powerful method for interrogating large data sets for the presence of sequence variants of interest. TASR is a fast, flexible and easy to use tool for targeted assembly.

## Introduction

The revolution in DNA sequencing technologies has enabled faster and cheaper data generation, to the point where data collection is becoming a less concerning bottleneck than data storage and analysis [1]. This is especially true for laboratories with limited informatics resources. TASR (Targeted Assembly of Sequence Reads) builds upon our previous SSAKE assembler [2] but, unlike its predecessor, it only considers reads for assembly that have a perfect 15 nt word match to input target sequences. Thus, TASR has particular utility for finding or confirming, through local assembly, the presence of specific sequences or sequence variants of interest. To our knowledge, de Bruijn graph assemblers published to date do not have this functionality. Here we demonstrate the utility of TASR for discriminating real from artifactual variant calls in tumour genomes, which may facilitate large-scale validation efforts. Further, we show that by targeted assembly it is possible to identify tumour-associated fusion transcripts and, finally, we demonstrate the utility of targeted assembly for identifying genome variations in ancient human DNA and large-scale human whole-genome sequencing projects.

## Results

### Algorithm

DNA sequence reads in a fastq or fasta format are fed into the algorithm via a file of filenames, using the –f option. DNA sequence targets, used to interrogate all raw reads in a sequence data set, are supplied as a multi fasta file using the –s option. Sequence targets are read first. From each target, every possible 15-character word from the plus and minus strand are extracted and stored in a hash table. Next, reads from the NGS data set are interrogated as described in [2], except that rather than using a greedy algorithm, any read with an exact match of its first 15 bases to any of the 15-mer words from the target sequence, is retained. These reads are collected in an array for subsequent assembly, thereby limiting the sequence space of the assembly to that of the target region. Note that low-complexity and large DNA sequence targets will draw in more reads, which will impact the performance of TASR. The choice of 15-mer is one that balances speed and specificity, and was used previously for targeted *de novo* assemblies of highly variable T cell receptor sequences [3], [4]. The identity and coverage of every base, within and beyond the user-provided target sequence, is stored in a hash table c. The sequence within the bounds of the user-supplied target sequence will exactly match the target itself, but recruited sequence reads will typically extend beyond the boundaries of the target sequence, and this flanking sequence may also be included in the assembly. In some instances the identity of the sequence that flanks the target may be unknown and may in fact be of greatest interest to the user. A consensus sequence is derived, taking exactly matching bases at each position within the target region, and extended outward, bi-directionally, to include the most represented base at positions outside the target sequence. In this regard, TASR is unchanged from the most recent version of SSAKE (v3.7) where consensus bases, situated outside the target region are derived using a majority-rule approach analogous to that of VCAKE [5]. For extension, each base has to be covered by user-defined -o (set to 2 by default) and its abundance relative to the next most called base equal or above the user-defined ratio –r (0.7). Extension is terminated when a position is encountered that does not meet these user-specified criteria. This process is repeated for each target sequence supplied in the –s file. TASR outputs target-derived contigs in fasta format, read positions and base coverage in text files and per-position information in a modified pileup format [6].

### Implementation

TASR is implemented in PERL and will run on any platform where PERL is installed. It is available (under GPL licence) from: http://www.bcgsc.ca/bioinfo/software/tasr and supplemental data files from: ftp://ftp.bcgsc.ca/supplementary/TASR.

### Testing

TASR runs were executed on a shared computer running CentOS 5.5, with 2 Intel Xeon X5680 CPUs at 3.33 Ghz (12 core, 24 threads) and 48 GB of RAM. At most, using our larger data set (3.5B Saqqaq Paleo-Eskimo NGS reads), TASR required <10 M RAM and ran for 12 hours.

### Verifying candidate SNVs in a lobular breast cancer genome

**Figure 1:**
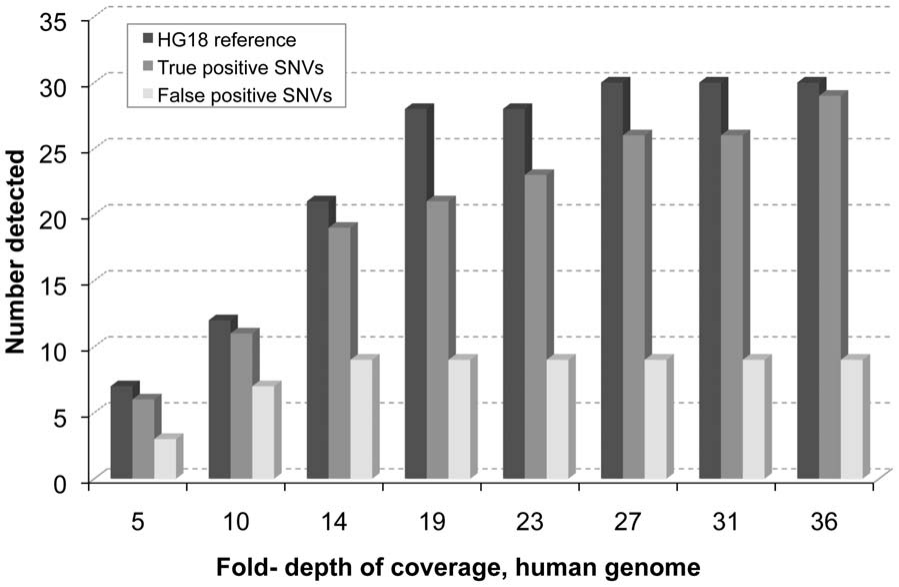
Detection of true positive versus false positive SNVs in lobular breast cancer. TASR was run incrementally on up to 2 billion, 51 and 76 nt lobular breast cancer NGS whole-genome shotgun reads, providing 5 to 36-fold coverage of the 3 Gbp human genome. We used as targets 51 nt sequences containing one of 31 SNVs detected by NGS read alignment and confirmed by Sanger sequencing (true positive), 31 matching sequences containing the reference base instead (reference) and 31 detected by NGS read alignment but not confirmed by Sanger sequencing (false positive). Although close to twice as much WGSS data had been generated from the LBC sample, we see that a fraction of that (∼19-fold) is sufficient for confirming most (68%) true positive SNVs.

Shah and co-workers [7] reported 32 confirmed somatic coding single nucleotide variants (SNVs) in a metastatic lobular breast cancer specimen. These confirmed variants resulted from testing, by capillary re-sequencing, of a larger number of putative variants that were originally suggested by whole tumour genome shotgun sequencing (WGSS) using the Illumina platform and running Maq [8] and SNVmix [9]. We interrogated 31 of these verified SNVs using 51 nt sequence targets containing either the mutant or the HG18 reference base. We also selected, at random, 31 SNVs that had been tested by capillary re-sequencing, but not verified (Shah et al., unpublished data). The sequence data, providing up to 36-fold coverage of the human genome, was analyzed incrementally (Figure 1) using TASR (default options). Maximum sensitivity was reached at moderate coverage (ca. 36-fold) where 29/31 (93.5%) of previously verified SNV were positive for the variant in question. Interestingly 30/31 (96.8%) also showed the reference base, reflecting the cellular heterogeneity of the tumour. At this same coverage only 9/31 of variants that failed previous verification by capillary sequencing showed the SNV, and of these, 7 showed the reference base. Thus, TASR does not re-identify the majority (>70%) of this subset of false-positive SNVs originally detected by Shah and colleague in NGS data, which highlights its utility as a tool to validate variants computationally, post sequence alignment, before undertaking orthogonal methods of variant verification such as resequencing. This improved level of discrimination for putative SNVs may significantly streamline on-going efforts to verify putative tumour mutations.

### Detecting an HPV fusion in HeLa cells

As part of the original development of NGS RNA-seq methodology, Morin and colleagues [10] evaluated HPV18 transcription in HeLa cell lines, which are known to carry this viral integration. We set out to determine the human-HPV fusion site by targeted assembly of these RNA-seq reads. Using four 38 nt sequence targets each comprising the same 37 HPV-specific bases preceding one of four possible DNA base as the 38^th^ base, respectively, we used TASR (default options) to interrogate 37.4 M RNA-seq reads. A single sequence target was extended into a contig that also comprised human cDNA sequences. Overall, 288 NGS reads co-assembled and 51 chimaeric reads, each having 1 or more HPV18- and human-only base(s) covered the fusion site.

### Detecting fusion transcripts in prostate adenocarcinoma

**Figure 2:**
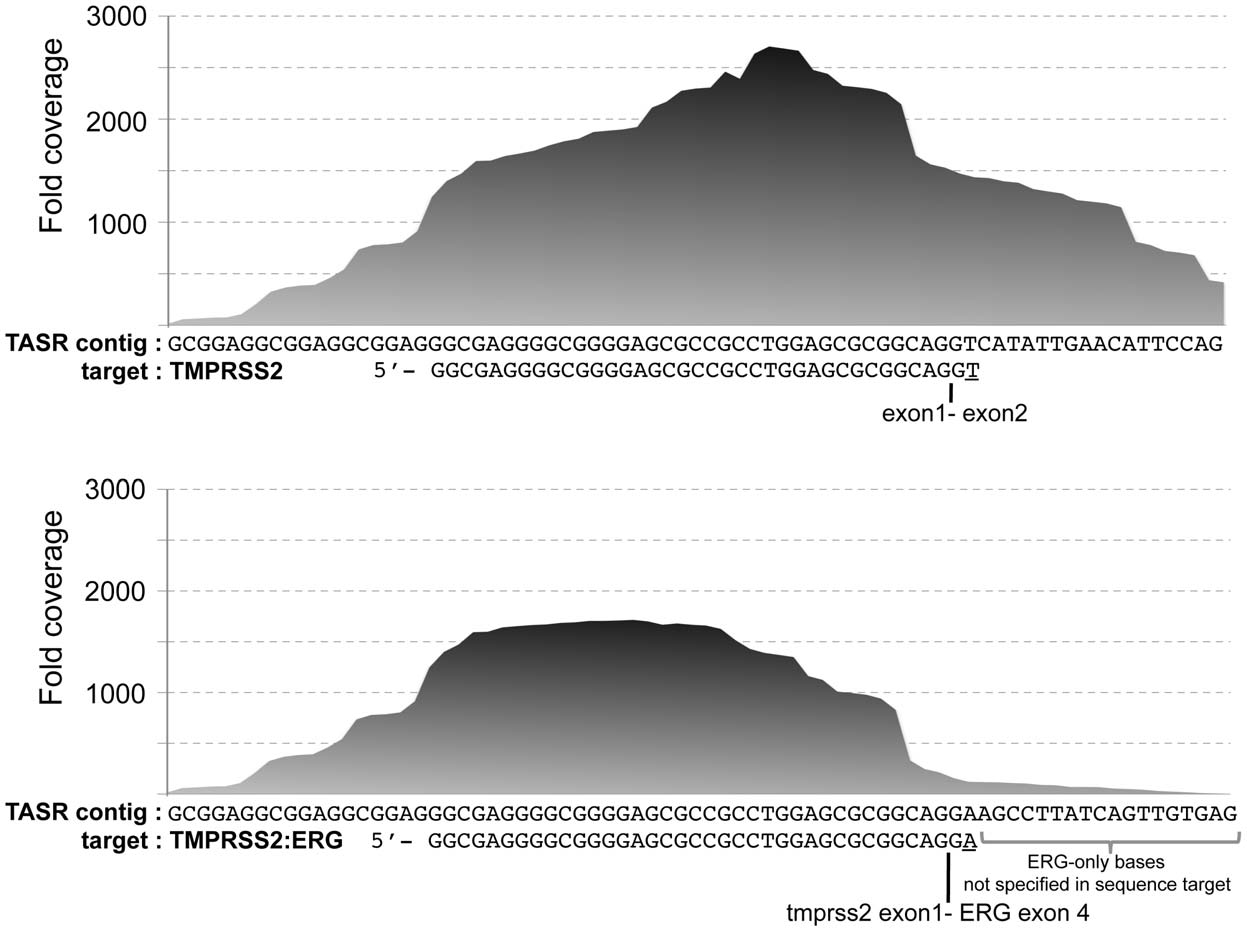
De novo assembly of prostate carcinoma RNA-seq data. Using a *TMPRSS2:ERG* target sequence that differs from a *TMPRSS2* target by a single base (underlined), TASR generated a contig, which captures 18 *ERG*-specific bases fused to exon 1 of *TMPRSS2* in a prostate adenocarcinoma sample (SRA accession SRX027125). These bases were not specified in the target sequence and thus, unknown from the original hypothesis. A total of 121 reads span the *TMPRSS2:ERG* fusion coordinate (underlined base). Higher base coverage is expected in the middle of the contig where 15-mer read recruitment reaches a maximum for both strand and is unaffected by the limiting effects of the minimum overlap (-m) option on the edge of the sequence target. This highlights the importance of using a sequence target that is sufficiently long and at least the same length as the input reads. From this result, it is very likely that the prostate adenocarcinoma sample contains an admixture of *TMPRSS2* transcripts, including the TMPRSS2{NM_005656.2}:r.1_71_ERG{NM_004449.3}:r.226_3097 fusion and that those have varied abundance, as reflected by high depth of coverage.

For each of the RNA-seq data sets corresponding to three adenocarcinoma and 3 non-tumour adjacent prostate tissue samples [11], we looked for the presence of a fusion gene, *TMPRSS2:ERG*, which is known to be common in prostate adenocarcinoma, and is a strong prognostic indicator [12]. Using two 50 nt target sequences, one containing the last 36 bp of exon 1 and the first 14 bp of exon 2 and the other the last 36 bp of *TMPRSS2* exon1 and first 14 bp of *ERG* exon 4, we ran TASR on each set (-m 15 –c 1 other options defaulted). In another experiment, we used two 38 nt sequence targets that differed only by their last 3′ base, simulating a scenario where very little information is available about a given event (Figure 2). We designed both experiments with the aim of detecting portion of the *TMPRSS2* and *TMPRSS2:ERG* transcripts and ran TASR under the same conditions. In both experiments, we found the fusion in 2/3 adenocarcinoma samples (SRX027124, SRX027125) and 1/3 adjacent normal sample (SRX027128) with reads spanning the fusion coordinate and containing both unique *ERG* and *TMPRSS2* bases. This finding, although unknown to us at the time of experimentation, is consistent with that of Nacu and colleagues who made this NGS data set public ahead of publication [11]. The *TMPRSS2*-only target also yields a contig for a non-fusion transcript. The number of fusion reads is generally lower than that of the *TMPRSS2* transcript (adenocarcinoma NGS data SRX027125, Figure 2) and may be an indicator of lower expression of the fusion, and/or cellular mosaicism. Although we used the entire available SRA data for each corresponding sample, we noticed that a single sequence run (e.g. SRR066437 ∼4.7 M spots) was sufficient for detection of the fusion in positive samples.

### Detecting SNPs in ancient human DNA

Rasmussen and colleagues [13] used a 4,000 year old sample of perma-frost-preserved hair to obtain the genome sequence of an early Paleo-Eskimo settler of Greenland, and reported single nucleotide polymorphisms (SNPs) including those known to confer phenotypes such as black hair color, dry ear wax, higher % fat mass, cold adaptation, not European light skin, and thick hair/shovel shaped upper front teeth. We interrogated the sequence data (3.5 billion 70 nt reads obtained from the SRA) to determine whether targeted assembly could recover these specific, but no other, SNPs. We ran TASR (default options) using sequence targets that were each 70 nt in length and contained the variant or reference alleles. By providing a comprehensive target sequence file that accounts for all known polymorphisms within the alleles tested, we hypothesized that only reads having the legitimate base will be recruited and co-assemble with the appropriate target sequence. We found read support for all six variants and no evidence of other alleles at these positions. Read coverage over each SNP is variable with the cold adaptation, high % fat mass, not European light skin, black hair, dry earwax and thick hair-associated polymorphisms covered by 3, 7, 7, 17, 22, 34 reads, respectively. None of the negative controls (sequence targets comprised of any possible alternate alleles from dbSNP) co-assembled sequence reads over the base under scrutiny.

### Detecting variations in a deeply sequenced trio from the 1000 Genomes pilot project

The 1000 Genomes Project Consortium has set out to map human genome variation by deep sequencing in order to gain insight into the relationship between genotypes and phenotypes [14]. In their pilot phase, the consortium analyzed low coverage whole-genome sequencing data from 179 individuals from 4 populations, plus deeply sequenced genomes from two trios (mother-father-daughter families) from Nigeria (YRI) and Utah (CEU), and reported variation. Here, from the YRI trio, we have randomly selected ten loss-of-function (LOF) SNPs, ten LOF indels, and ten structural variants (SVs) from chromosomes 1 and 2 that were non-LOF deletions (> = 50 nt) and re-assessed these using TASR. For SNPs and indels, we used the dbSNP accession numbers provided by the consortium to retrieve ∼52 nt-long target sequences. For the re-identification of SVs, target sequences varied in length from 58–90 nt and their design was informed by the vcf file summarizing base deletions > = 50 nt identified from the YRI family data (trio.2010_10.deletions.sites.vcf). A subset of the raw sequence reads were obtained which provided approximately 27-fold average genome coverage for each subject (∼13.6× diploid coverage). TASR was run on each set (options defaulted) and the results are summarized in Table 1. Out of 30 total genome variations inspected, 29 (97%) were re-identified by TASR in at least one member of the trio. The mother had the lowest re-identification rate at 90%. Complete variant re-identification was not expected since we used only a subset of the raw data. Further, TASR's stringency is very high and sequences containing error or adjacent variants not contained within the target sequence will impair read recruitment and assembly. Yet, interesting observations can be made. For instance, when comparing TASR variant re-identification to the portion of the 1000 genomes data with available genotype information for all three individuals (SNP and indels), we obtain a 93.3% concordance. Further 9/10 SNPs, 7/10 indels and 8/10 SVs are re-identified in all family members in a manner consistent with the family member genotypes (when applicable) and there are no instances of variants that are inconsistent with inheritance. For example, 20/20 SNP and indel detected by TASR were present in the child and one of the two parents, and transmission can be inferred for these variants.

**Table 1:** Re-identification of human genome variations from the 1000 Genomes pilot project.

Genome variations			Read count(s) over SNP or junction^1^ and genotype^2^ for YRI trio member		
Type	Identifier(dbSNP or 1000 Genomes ID)	SNP	Mother	Father	Daughter
SNP	rs1736565	C/T	0/7^T/T^	11/9^C/T^	0/15^T/T^
	rs6443930	C/G	7/5^C/G^	12/0^C/C^	14/0^C/C^
	rs2645341	C/T	0/5^T/T^	0/17^T/T^	0/9^T/T^
	rs13191323	C/T	0/89^T/T^	0/11^T/T^	0/15^T/T^
	rs1965370	C/G	0/11^G/G^	0/14^G/G^	0/9^G/G^
	rs10862125	C/T	0/12^T/T^	0/11^T/T^	0/14^T/T^
	rs6511602	C/T	0/0^T/T^	0/4^T/T^	0/7^T/T^
	rs2245425	G/A	2/2^G/A^	9/2^G/A^	12/7^G/A^
	rs4509745	C/T	5/6^C/T^	8/10^C/T^	0/21^T/T^
	rs7004273	G/A	0/10^A/A^	0/13^A/A^	0/15^A/A^
Indels	rs58432514	−/G	0/1^G/G^	0/6^G/G^	0/15^G/G^
	rs11450450	−/C	0/2^C/C^	0/10^C/C^	0/19^C/C^
	rs35933224	−/TTTG	6/0^−/TTTG^	17/46^−/TTTG^	10/0^−/−^
	rs140511	−/C	0/1^C/C^	0/4^C/C^	0/8^C/C^
	rs11382443	−/A	9/0^−/−^	6/8^−/A^	5/3^−/A^
	rs57304020	−/G	0/3^G/G^	0/2^G/G^	0/5^G/G^
	rs3078330	−/TA	0/2^TA/TA^	0/11^TA/TA^	0/10^TA/TA^
	rs11303415	−/C	1/0^−/−^	3/0^−/C^	2/3^−/C^
	rs59393160	−/GT	0/7^−/GT^	0/14^GT/GT^	0/15^GT/GT^
	rs35117663	−/AG	5/2^−/AG^	23/0^−/−^	11/0^−/−^
SV	P2_M_061510_1_103	−/Δ175G	4/0^3^	4/8	12/1
	P2_M_061510_1_308	−/Δ58A	0/3	8/0	0/10
	P2_M_061510_1_533	−/Δ67G	11/0	7/11	10/0
	P2_M_061510_1_198	−/Δ66T	0/3	5/5	11/6
	P2_M_061510_2_234	−/Δ103*x* 4	0/0	7/3	11/6
	P2_M_061510_2_875	−/Δ60C	0/0	0/0	0/0
	P2_M_061510_2_606	−/Δ76C	0/12	15/4	10/14
	P2_M_061510_2_578	−/Δ63C	5/0	9/0	18/0
	P2_M_061510_2_858	−/Δ63ATCATA	0/4	1/0	9/8
	P2_M_061510_2_210	−/Δ61CTCAT	7/10	7/0	14/0

SNP: Single Nucleotide Variant SV: Structural Variation.

1 Re-identified by TASR. Only reads spanning variation within 5 bases of read start/end were counted.

2 In superscript, genotypes were determined by the 1000 genomes project. This information is not available for SVs.

3 A/B : A = coverage of reads over the non-deleted portion B = coverage over the deletion breakpoint.

4 *x* = ACTAGTGCATTTCAATAATCATG.

Underlined are discrepancies between TASR and the genotype calls, all of which are due to insufficient read coverage.

## Discussion

TASR is a targeted *de novo* assembler that uses supplied sequences to target initial read recruitment and assembly. The targets can be any sequence, actual read, existing reference or synthetic sequence. For example, when testing for fusion transcripts, or any other unknown sequence flanking a target, NGS data sets could be interrogated using four distinct target sequences, each with one of the four possible nucleotides as their last 3′ base. This is a key advantage of targeted assembly, since alignment of NGS sequence to a complete reference would not be expected to return sequence reads that contained a significant number of non-reference bases. Likewise, reads representing a rare fusion or insertion event may be excluded from whole genome *de novo* assemblies if they have low representation in the NGS raw data. Further, most large-scale *de novo* assemblies are precluded by the shear data size, such as those processed in our study (e.g. 3.5B Saqqaq Paleo-Eskimo NGS reads from 238 Illumina sequence lanes). Although the development of *de novo* assemblers such as ABySS [15] and SOAPdenovo [16], now makes whole-genome and whole-transcriptome human NGS read assembly a reality, researchers would still have to sift through thousands, if not millions, of contigs for sequences of interest. We provide a solution that allows relatively quick (∼3.5B reads from 238 sequencing lanes providing 81× average coverage of a 3 GB human genome, processed in 12 hrs) and flexible hypothesis testing that is targeted to a genomic region of interest, whether it comprises a fusion, translocation, SNV or SNP.

In TASR, the assembly results are influenced strongly by the design of the target sequences used as input. Targets that are as long as the shortest read being considered and shorter than two read sizes (e.g. a 70 bp target could be extended by 2 * (70 – m option) will produce the best results. This length will ensure that all overlapping 15-mer from a given target recruit the maximum number of candidate reads for assembly. Also, the use of longer NGS reads (>70) will increase the chance of generating longer contigs with more novel or previously unknown bases, which is instrumental in the characterization of previously unknown events, such as the detection of a viral integration site. For example, using 33 nt NGS reads and setting the minimum overlap to 15 could extend the target on each side by at most 18 bp (33-15) whereas the use of 150 nt reads with 150 nt targets could yield 420 bp contigs having up to 270 previously uncharacterized bases.

Frequently, it may be of interest to mine a NGS data set for the presence of one or more single nucleotide variants of interest; for example, variants that have known associations to specific traits or genetic disorders. For SNV or SNP detection, it is prudent to have the base under scrutiny in the middle of the target, to increase the chances of recruiting candidate reads that have the particular base at any possible position. It may seem peculiar to use a *de novo* assembler to help validate single-base changes in genomes, especially now that fast and large-scale read alignment methods, such as bwa [17], exist. TASR has the advantage of 1) conducting targeted assembly to a specific region and thus, alleviate the need of sifting through large alignment or assembly files. Also, 2) it is very stringent in that it will only recruit and co-assemble NGS reads whose bases overlapping the target sequence are in perfect agreement. This has the advantage of rapidly testing a simple hypothesis such as whether a locus has the reference base, a variant or both, by looking at a read pileup over the base under scrutiny. Lastly, 3) it performs *de novo* assemblies, such that overlapping bases that fall outside the target region have the potential to characterize a novel sequence.

For targeted sequence assemblies, the 1000 Genomes Project Consortium [14] employed TIGRA (L. Chen, unpublished: http://genome.wustl.edu/software/tigra_sv) to reassemble NGS reads that had been initially aligned to a reference. TASR is fundamentally different in that it does not require *a priori* whole-genome short read alignment for targeted assembly and, as such, will interrogate all raw reads supplied as input for the presence of the variant. The difference between *ab initio* variant discovery by alignment-guided targeted assemblers and variant re-identification with TASR is one that highlights the utility of the latter for genotyping directly from raw NGS data, without the need for whole-genome alignments. This method has the advantage of considering all reads for assembly with overlap potential to target sequences, including those that may otherwise not align to a specific site and excluding those that may have been misaligned to a reference when using alignment-guided assembly approaches. A recent analysis [18] has shown that considerable novel human genome sequences remain to be discovered. While the haploid human genome size is ∼3.156 Gbp (GRCh37.p3; http://www.ncbi.nlm.nih.gov/genome/assembly/grc/human/data/index.shtml), the human pan-genome may have an estimated 19 to 40 Mb (up to 1.3%) of novel sequences, not found in the reference human genome [18]. It should be noted that TASR is not suited for the *ab initio* characterization of novel, kilobase size, sequence segments for which no information is available beforehand. This is accomplished best by whole-genome *de novo* assembly methods [16], [18] and alignment-guided targeted assemblies. However, while TASR was not designed for the purpose of comprehensive pan-genome analysis, it may have utility in searching population data sets for sequences that are unique, putatively, to an individual.

The utility of TASR is to interrogate specific target sequences by local assembly. The targets can be any sequence, an actual read, a reference or synthetic sequence. Here we demonstrate, using TASR, the mining of NGS data sets for fusion transcripts and two types of single nucleotide variants (SNV), somatic mutations in tumour genomes and polymorphisms in ancient DNA and whole-genome sequence projects. TASR uses a stringent targeted assembly scheme, where the more complex and unique a target sequence is, the less likely non-specific reads are to co-assemble, facilitating variant detection. TASR may be used in a manner that is complementary to NGS read alignment tools, in order to help confirm false-positive events that may result from NGS sequencing or mapping errors. As it performs a *de novo* assembly of reads outside the target region, it may be used for the targeted assembly of chimaeric reads whose bases may help characterize novel fusion, translocation or integration events. Since TASR does not require indexed databases or multi-staged runs it is easy to use. It runs on commodity hardware with a low computer resource footprint.

## Methods

Lobular breast cancer (LBC) whole-genome sequence data were previously described [7]. We processed ∼2B paired-end reads (76 and 51 bp) which provided ∼36-fold coverage of the human genome. The HeLa RNA-seq data (37.4 M single-end 31 bp reads) was obtained from Morin and colleagues [10]. RNA-seq data from 3 human prostate adenocarcinoma and 3 matched adjacent normal samples was obtained from the SRA (SRP003611) [11]. An average of 32 M reads (33 bp) was downloaded for each of 5 samples, and from the 6th sample, SRX027125, we obtained 65.5 M reads. All of the 3.5B whole-genome shotgun sequences (70 bp reads in 238 fastq files totalling 878 Gbytes) from the extinct Saqqaq Paleo-Eskimo [13] was obtained from the SRA (SRP001453), for the purpose of targeted assembly of SNPs (dbSNP: rs5746059, rs17822931, rs16891982, rs1426654, rs3827760, rs1042522). From the 1000 Genomes FTP site [14] (ftp://ftp.ncbi.nlm.nih.gov/1000genomes/ftp/pilot_data/data/), an average of 2.1×10^9^ whole-genome NGS fastq reads for each of the mother (NA19238), father (NA19239) and daughter (NA19240) YRI trio sample were downloaded. A total of sixty sequence targets containing the ancestral or variant allele were designed from ten randomly selected SNPs (dbSNP: rs1736565, rs6443930, rs2645341, rs13191323, rs1965370, rs10862125, rs6511602, rs2043336, rs1128966, rs7004273), indels (dbSNP: rs58432514, rs11450450, rs35933224, rs140511, rs1138244, rs57304020, rs5794199, rs3078330, rs10591060, rs11340767) and ten SVs that were deletions (> = 50 nt) located on chromosomes 1 and 2 (P2_M_061510: 1_103, 1_308, 1_533, 1_198, 2_234, 2_875, 2_606, 2_578, 2_858, 2_210).
